# Cross-Coupling Synthesis of Methylallyl Alkenes: Scope Extension and Mechanistic Study

**DOI:** 10.3390/molecules201219886

**Published:** 2015-12-21

**Authors:** Clémence Tabélé, Christophe Curti, Youssef Kabri, Nicolas Primas, Patrice Vanelle

**Affiliations:** Aix-Marseille Université, CNRS, ICR, UMR 7273, Laboratoire de Pharmaco-Chimie Radicalaire, Faculté de Pharmacie, 27 Boulevard Jean Moulin–CS30064, 13385 Marseille cedex 05, France; clemence.tabele@ap-hm.fr (C.T.); christophe.curti@univ-amu.fr (C.C.); youssef.kabri@univ-amu.fr (Y.K.); nicolas.primas@univ-amu.fr (N.P.)

**Keywords:** palladium, cross-coupling reactions, boronic acids, allyl alcohol

## Abstract

Cross-coupling reactions between 2-methyl-2-propen-1-ol and various boronic acids are used to obtain aromatic-(2-methylallyl) derivatives. However, deboronation or isomerization side reactions may occur for several boronic acids. We describe herein the synthesis of original alkenes with good yields under mild reaction conditions that decrease these side reactions. The scope of this environmentally benign reaction is thereby extended to a wide variety of boronic acids. A mechanistic study was conducted and suggested a plausible catalytic cycle mechanism, pointing to the importance of the Lewis acidity of the boronic acid used.

## 1. Introduction

Recently, we described an environmentally friendly way to synthesize 2-methylallyl alkenes via cross-coupling reactions between arylboronic acids and 2-methyl-2-propen-1-ol used as both a reactant and the solvent [1]. The results suggested that this kind of reaction could easily be extended to various allyl alcohols under our reaction conditions. This new process shows a high functional group tolerance and is reproducible for a wide range of arylboronic acids. 

These promising results, coupled with the need for new terminal alkenes for the synthesis of potentially bioactive compounds [2,3], encouraged us to extend this protocol to other boronic acids. While the yields obtained were often good, in several cases, secondary reactions occurred. The results of a mechanistic study elucidating this original cross-coupling reaction are presented herein as a complement to our existing work [1].

## 2. Results and Discussion

### 2.1. Reaction Conditions Optimization

**Figure 1:**
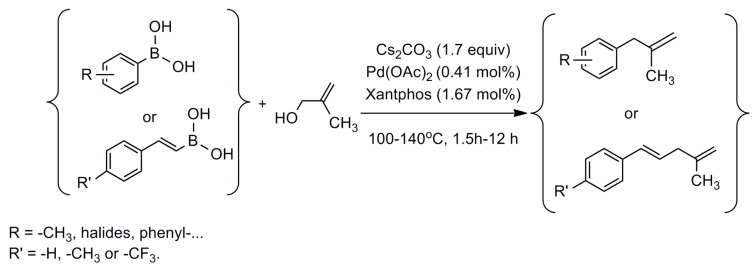
Cross-coupling reactions of 2-methyl-2-propen-1-ol with boronic acids.

Reaction conditions previously described were optimal for a wide variety of boronic acids (Figure 1). However, for some, we failed to obtain the corresponding allyl alkene in good yields. Results are presented in Table 1.

Excellent to quantitative yields were obtained with m-substituted and p-substituted arylboronic acids (**2**, **5**, **6**, **7** and **11**). Deboronation side reactions were observed for most of the vinylic or the o-substituted boronic acids (**1**, **4**, **12** and **13**) when cross-coupling reactions were performed at 140 °C, lowering the yields. Moreover, the same deboronation outcome was observed with [2-fluoro-(1,1′-biphenyl)-4-yl]boronic acid, leading to product **8** in a moderate yield. Alkene **3** was obtained in low yield (52%), as we observed the occurrence of the isomerization side reaction, providing product **3′**. Lower yields due to isomerization side reactions were also observed with methoxy-substituted boronic acids (products **9** and **10**), providing products **9′** and **10′**.

**Table 1:** Yields obtained for cross-coupled products.

Entry	Alkenes	Side Product	Yield (%) ^1,2^	
1	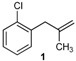		1	51
2	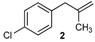		2	95
3	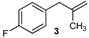	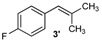	3/3′	52/38
4	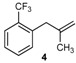		4	55
5	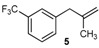		5	77
6	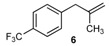		6	78
7	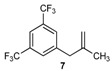		7	80
8	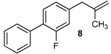		8	57
9	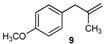	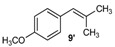	9/9′	45/30
10	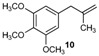	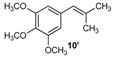	10/10′	41/42
11	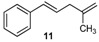		11	77
12	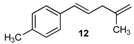		12	50
13	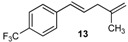		13	46

^1^ Reaction conditions: MW (microwave irradiation) in a sealed reactor, 140 °C, 1.5 h, air (Condition a). ^2^ Isolated yields.

To deal with these various cross-coupling problems, parameters were refined. Results obtained are presented in Table 2.

**Table 2:** Yield optimization for cross-coupled products.

Entry	Alkenes	Yield (%) ^1^
14	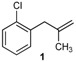	61 ^2^
15	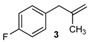	74 ^3^
16	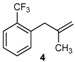	97 ^3^
17	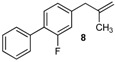	78 ^3^
18	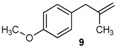	97 ^3^
19	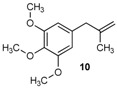	99 ^2^
20	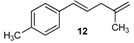	92 ^3^
21	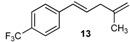	88 ^3^

^1^ Isolated yields. ^2^ Reaction conditions: 100 °C, 12 h, air (Condition c). ^3^ Reaction conditions: MW in a sealed reactor, 120 °C, 1 h, air (Condition b).

Deboronation reactions have been widely described [4,5], with many solutions studied [6]. Milder reaction conditions were considered to reduce this side reaction. Therefore, reaction conditions were slightly modified (120 °C for 1 h instead of 140 °C for 1.5 h), dramatically enhancing yields for products **3**, **4**, **8**, **9**, **12** and **13**. Unfortunately, yields were not significantly modified for products **1** and **10** under these experimental conditions. To overcome deboronation and isomerization, reactions were carried out under normal heating conditions for 12 h [7]. Under these new milder conditions, problems of deboronation and isomerization were reduced for compounds **1** and **10**, respectively. The yields were improved from 51% to 61% for **1** and from 41% to near quantitative for **10**. However, compound **1** was observed to be very sensitive to the air and consequently needs to be kept under nitrogen atmosphere to avoid degradation. Under these optimized reaction conditions, isomerization products were not identified for products **3**, **9** and **10**. 

Experimental conditions were also tested for a boronic acid leading to alkene with good yields under classical conditions. Starting from *p*-chloroboronic acid, the corresponding alkene was obtained with excellent yield (95%, Table 1, Entry 2) under condition a (MW (microwave irradiation) in a sealed reactor, 140 °C, 1.5 h, air). Alkene yields decreased to 87% under condition b (MW in a sealed reactor, 120 °C, 1 h, air) and 80% under condition c (100 °C, 12 h, air). 

With regard to (*E*)-styrylboronic acids, the NMR assessment showed that the stereochemistry of cross-coupled products is maintained, providing only (*E*)-isomers (**11**, **12**, **13**), as opposed to previous reports [8]. Such stereoselectivity argues against an addition/elimination pathway of this cross-coupling reaction [9].

Furthermore, under the different experimental conditions (conditions a, b and c) and for all reactions, formation of a biphasic system was observed after the cross-coupling reactions. The bottom phase was identified as small quantities of water.

### 2.2. Mechanistic Study

**Figure 2:**
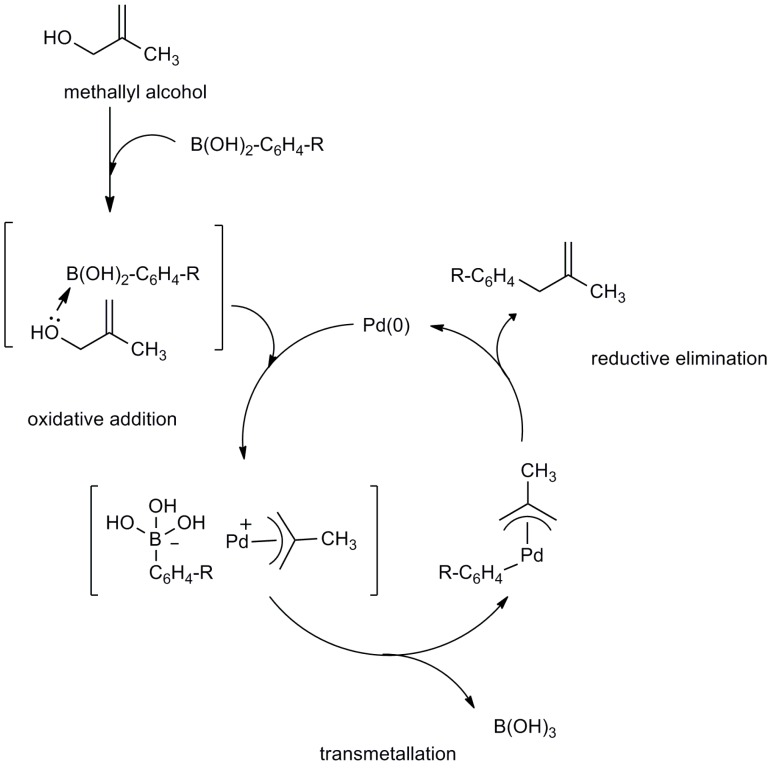
Plausible catalytic cycle mechanism.

To identify a plausible mechanism [10] for the cross-coupling reaction of methallyl alcohol with arylboronic acids under our reaction conditions (Figure 2), further reactions were carried out.

**Figure 3:**
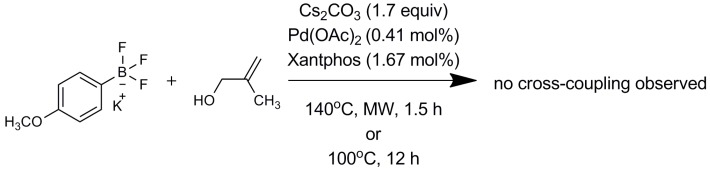
Reactions of 2-methyl-2-propen-1-ol with potassium phenyltrifluoroborate.

First, assays were carried out with boronate anions such as potassium phenyltrifluoroborate. The cross-coupling reaction was inefficient (Figure 3) and no coupled products were obtained, showing that the Lewis acidic features of organoboronic acid derivatives are required. Strong reaction conditions led to complete deboronation of the substrate.

This finding supports the catalytic cycle mechanism hypothesis implying that the boron atom needs to be free enough to interact with the -OH of methallyl alcohol, as shown in Figure 2.

These results are consistent with the role of the base in our cross-coupling reaction conditions. When the base was suppressed from our protocol, the cross-coupling reaction was not observed [1]. Previous studies on cross-coupling reactions of allylic alcohols with boronic acids did not report the addition of a base in aprotic solvents such as CH_2_Cl_2_, THF, toluene or dioxane [11,12]. On the other hand, when these reactions were launched in a protic solvent such as water, a catalytic amount of base was required to increase reactivity [13,14]. In our protocol, methallyl alcohol, previously described as an unreactive substrate [10], acts both as a reagent and a solvent. Cesium carbonate could react with methallyl alcohol, thus increasing its basicity and its potential Lewis interaction with the boron atom.

**Figure 4:**
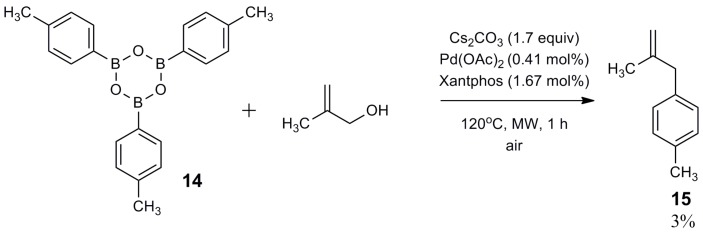
Synthesis of compound **15** from boroxine **14**.

Next, we explored boroxine reactivity. A cross-coupling reaction between boroxine **14** and methallyl alcohol was carried out (reaction condition b) (Figure 4). Compound **15** was obtained in very low yield, showing that boroxine under anhydrous conditions is not a good substrate for the cross-coupling reaction. Under the same experimental conditions, when three equivalents of water were added, compound **15** was obtained in good yield (90%).

**Figure 5:**
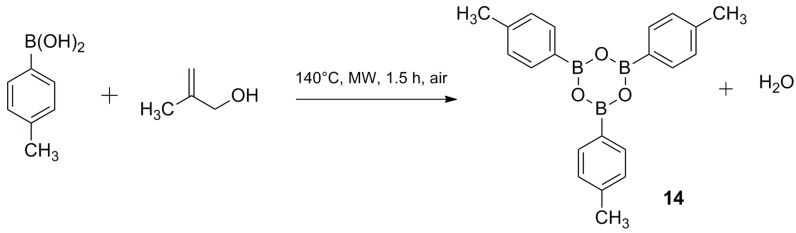
Conversion of boronic acid to boroxine when heating.

Moreover, although water formation was observed in all our cross-coupling reactions, it was not observed when boroxin was used as a reagent. This water could come from methallyl alcohol via a dehydrative pallado-catalyzed mechanism [15], but we hypothesized instead that it comes from the dehydration of three equivalents of boronic acid to one equivalent of boroxine. This reaction can provide three equivalents of water apparently without decreasing reactivity, according to previous studies [16]. In order to determine whether water formed in cross-coupling reactions is due to dehydration of boronic acid, we carried out a reaction of *p-*tolylboronic acid in methallyl alcohol: the formation of 4-methylphenylboroxine was observed with a boronic acid conversion to boroxine of 43% (Figure 5).

Yields from cross-coupling reactions may be directly linked to the conversion of boronic acid to boroxine [17]; this reversible conversion appears to be connected with the ability of the boronic acid to release excess water. As water formation was not observed during the cross-coupling reaction of boroxin, the water observed in cross-coupling reactions with classic boronic acids was only due to dehydration into boroxine: it proves that boronic acid was converted to boroxin under our experimental conditions. This equilibrium and the low reactivity of boroxin could explain the variable yields of the cross-coupled products obtained, depending on the boronic acid used.

## 3. Materials and Methods

### 3.1. General Information

TLC were performed on 5 cm × 10 cm aluminum plates coated with silica gel (layer 0.2 mm) 60 F 254 (Merck, Darmstadt, Germany) in an appropriate solvent. Boiling points were determined through capillary tubes, with a B-540 Büchi melting point apparatus, at 760 mmHg. The ^1^H-NMR and ^13^C-NMR spectra were recorded in CDCl_3_ or DMSO, with tetramethylsilane (Me_4_Si) as an internal reference using a Bruker ARX 200 spectrometer operating at 200 MHz for ^1^H-NMR and 50 MHz for ^13^C-NMR; a Bruker Avance III nanobay-300 MHz spectrometer operating at 300 MHz for ^1^H-NMR and 75 MHz for ^13^C-NMR; and a Bruker Avance III nanobay-400 MHz spectrometer operating at 400 MHz for ^1^H-NMR and 101 MHz for ^13^C-NMR; spectra were carried out at the Service Interuniversitaire de RMN de la Faculté de Pharmacie de Marseille and at the Spectropole de la Faculté des Sciences site Saint-Jérôme. The ^1^H chemical shifts are quoted in parts per million as δ downfield from tetramethylsilane (δ 0.00) as an internal standard and the ^13^C chemical shifts were referenced to the solvent peaks: CDCl_3_ (76.9 ppm) or DMSO-*d*_6_ (39.6 ppm). Coupling constants (*J* values) are given in hertz. NMR multiplicities are abbreviated as follows: s (singlet), d (doublet), t (triplet), q (quartet) and m (a more complex multiplet or overlapping multiplets). Microwave-assisted reactions were performed in a monomode microwave oven for the Suzuki-Miyaura cross-coupling reactions (Biotage Initiator Microwave oven using 10–20 mL or 2–5 mL sealed vials; temperatures were measured with an IR-sensor and reaction times given as hold times). Elemental analysis and mass spectra, run on an API-QqToF mass spectrometer, were carried out at the Spectropole de la Faculté des Sciences site Saint-Jérôme. All commercial reagents were used without purification.

### 3.2. Synthesis of Methylallyl Alkenes

A solution of 2-methyl-2-propen-1-ol (5 mL), boronic acid (4.1 mmol, 1 equiv.), palladium(II) acetate (3.8 mg, 0.017 mmol, 0.0041 equiv.), Xantphos (38 mg, 0.068 mmol, 0.0167 equiv.) and cesium carbonate (2.3 g, 7 mmol, 1.7 equiv.) was stirred in a sealed vial for 1.5 h at 140 °C, under microwave irradiation (reaction conditions **a**) or for 1 h at 120 °C, under microwave irradiation (reaction conditions **b**) or for 12 h at 100 °C, under classical heating (reaction conditions **c**). Then the solid residue was filtered off. The filtrate was purified by fractional distillation under atmospheric pressure on a Vigreux column (first fraction: 2-methyl-2-propen-1-ol, b.p. 115 °C, second fraction: alkene with reported b.p.) to obtain desired alkene.

*1-Chloro-2-(2-methylallyl)benzene* (**1**): obtained from (2-chlorophenyl)boronic acid (641 mg) using method c, as a colorless oil (420 mg, 61%), b.p. 190 °C. ^1^H-NMR (CDCl_3_, 200 MHz): δ = 7.49–7.09 (m, 4H, 4 Ar-H), 4.62 (s, 1H, CH_2_), 4.86 (s, 1H, CH_2_), 3.47 (s, 2H, CH_2_), 1.77 (s, 3H, CH_3_). ^13^C-NMR (CDCl_3_, 75 MHz): δ = 143.5 (C), 137.4 (C), 134.5 (C), 131.0 (CH), 129.5 (CH), 127.6 (CH), 126.7 (CH), 112.3 (CH_2_), 41.4 (CH_2_), 22.5 (CH_3_). HRMS (ESI +) *m*/*z* calcd for C_10_H_11_Cl [M]^+^: 166.0549. Found: 166.0585.

*1-Chloro-4-(2-methylallyl)benzene* (**2**) [18]: obtained from (4-chlorophenyl)boronic acid (641 mg) using method a, as a colorless oil (650 mg, 95%), b.p. 229 °C. ^1^H-NMR (CDCl_3_, 200 MHz): δ = 7.26 (d, ^3^*J*_H-H_ = 8.4 Hz, 2H, 2 Ar-H), 7.12 (d, ^3^*J*_H-H_ = 8.3 Hz, 2H, 2 Ar-H), 4.73 (s, 1H, CH_2_), 4.83 (s, 1H, CH_2_), 3.29 (s, 2H, CH_2_), 1.67 (s, 3H, CH_3_). ^13^C-NMR (CDCl_3_, 75 MHz): δ = 144.6 (C), 138.2 (C), 131.9 (C), 130.3 (2 CH), 128.4 (2 CH), 112.3 (CH_2_), 44.0 (CH_2_), 22.0 (CH_3_). HRMS (ESI +) *m*/*z* calcd for C_10_H_11_Cl [M]^+^: 166.0549. Found: 166.0914.

*1-Fluoro-4-(2-methylallyl)benzene* (**3**) [19]: obtained from (4-fluorophenyl)boronic acid (574 mg) using method b, as a yellow oil (450 mg, 74%), b.p. 186 °C. ^1^H-NMR (CDCl_3_, 200 MHz): δ = 7.22–6.84 (m, 4H, 4 Ar-H), 4.72 (s, 1H, CH_2_), 4.82 (s, 1H, CH_2_), 3.30 (s, 2H, CH_2_), 1.83 (s, 3H, CH_3_). ^13^C-NMR (CDCl_3_, 75 MHz): δ = 161.5 (d, ^2^*J*_C-F_ = 243.6 Hz, C), 140.8 (C), 135.3 (d, ^5^*J*_C-F_ = 3.2 Hz, C), 130.3 (d, ^4^*J*_C-F_ = 7.8 Hz, 2 CH), 115.0 (d, ^3^*J*_C-F_ = 21.1 Hz, 2 CH), 112.1 (CH_2_), 42.3 (CH_2_), 21.0 (CH_3_). HRMS (ESI +) *m*/*z* calcd for C_10_H_11_F [M]^+^: 150.0845. Found: 150.0893.

*1-Fluoro-4-(2-methylprop-1-en-1-yl)benzene* (**3′**) [20]: obtained from (4-fluorophenyl)boronic acid (574 mg) using method a, as a yellow oil (316 mg, 52%), b.p. 158 °C. ^1^H-NMR (CDCl_3_, 200 MHz): δ = 7.24–6.88 (m, 4H, 4 Ar-H), 6.13 (s, 1H, CH), 1.85 (s, 6H, CH_3_). ^13^C-NMR (CDCl_3_, 75 MHz): δ = 161.8 (C), 140.8 (C), 135.6 (C), 130.6 (2 CH), 125.6 (CH), 21.0 (CH_3_), 19.6 (CH_3_).

*1-(2-Methylallyl)-2-(trifluoromethyl)benzene* (**4**): obtained from [2-(trifluoromethyl)phenyl]boronic acid (779 mg) using method b, as a colorless oil (800 mg, 97%), b.p. 154 °C. ^1^H-NMR (CDCl_3_, 200 MHz): δ = 7.81–7.24 (m, 4H, 4 Ar-H), 4.59 (s, 1H, CH_2_), 4.89 (s, 1H, CH_2_), 3.51 (s, 2H, CH_2_), 1.71 (s, 3H, CH_3_). ^13^C-NMR (CDCl_3_, 75 MHz): δ = 144.1 (C), 140.6 (C), 132.3 (CH), 130.6 (q, ^3^*J*_C-F_ = 32.0 Hz, C), 128.7 (CH), 125.6 (q, ^4^*J*_C-F_ = 3.7 Hz, CH), 124.3 (d, ^2^*J*_C-F_ = 272.3 Hz, C), 123.0 (q, ^4^*J*_C-F_ = 3.8 Hz, CH), 112.8 (CH_2_), 44.3 (CH_2_), 22.0 (CH_3_). HRMS (ESI +) *m*/*z* calcd for C_11_H_11_F_3_ [M]^+^: 200.0813. Found: 200.0857.

*1-(2-Methylallyl)-3-(trifluoromethyl)benzene* (**5**): obtained from [3-(trifluoromethyl)phenyl]boronic acid (779 mg) using method a, as a colorless oil (630 mg, 77%), b.p. 167 °C. ^1^H-NMR (CDCl_3_, 200 MHz): δ = 7.56–7.30 (m, 4H, 4 Ar-H), 4.75 (s, 1H, CH_2_), 4.86 (s, 1H, CH_2_), 3.38 (s, 2H, CH_2_), 1.68 (s, 3H, CH_3_). ^13^C-NMR (CDCl_3_, 75 MHz): δ = 144.1 (C), 140.6 (C), 132.3 (CH), 130.6 (q, ^3^*J*_C-F_ = 31.7 Hz, C), 128.7 (CH), 125.6 (q, ^4^*J*_C-F_ = 3.8 Hz, CH), 124.2 (q, ^2^*J*_C-F_ = 272.2 Hz, C), 123.0 (q, ^4^*J*_C-F_ = 3.9 Hz, CH), 112.8 (CH_2_), 44.3 (CH_2_), 22.0 (CH_3_). HRMS (ESI +) *m*/*z* calcd for C_11_H_11_F_3_ [M]^+^: 200.0813. Found: 200.1175.

*1-(2-Methylallyl)-4-(trifluoromethyl)benzene* (**6**): obtained from [4-(trifluoromethyl)phenyl]boronic acid (779 mg) using method a, as a yellow oil (630 mg, 78%), b.p. 160 °C. ^1^H-NMR (CDCl_3_, 200 MHz): δ = 7.56 (d, ^3^*J*_H-H_ = 8.1 Hz, 2H, 2 Ar-H), 7.31 (d, ^3^*J*_H-H_ = 8.0 Hz, 2H, 2 Ar-H), 4.76 (s, 1H, CH_2_), 4.87 (s, 1H, CH_2_), 3.38 (s, 2H, CH_2_), 1.69 (s, 3H, CH_3_). ^13^C-NMR (CDCl_3_, 75 MHz): δ = 144.1 (C), 141.3 (C), 129.2 (2 CH), 128.5 (q, ^3^*J*_C-F_ = 32.4 Hz, C), 125.2 (q, ^4^*J*_C-F_ = 3.8 Hz, 2 CH), 124.4 (q, *^2^J*_C-F_ = 271.8 Hz, C), 112.7 (CH_2_), 44.4 (CH_2_), 22.0 (CH_3_). HRMS (ESI +) *m*/*z* calcd for C_11_H_11_F_3_ [M]^+^: 200.0813. Found: 200.1176.

*1-(2-Methylallyl)-3,5-bis(trifluoromethyl)benzene* (**7**): obtained from [3,5-bis(trifluoromethyl)phenyl]boronic acid (600 mg) using method a, as a colorless oil (500 mg, 80%), b.p. 166 °C. ^1^H-NMR (CDCl_3_, 200 MHz): δ = 7.74 (s, 1H, Ar-H), 7.65 (s, 2H, 2 Ar-H), 4.76 (s, 1H, CH_2_), 4.90 (s, 1H, CH_2_), 3.44 (s, 2H, CH_2_), 1.75 (s, 3H, CH_3_). ^13^C-NMR (CDCl_3_, 75 MHz): δ = 143.0 (C), 142.3 (C), 131.5 (q, ^3^*J*_C-F_ = 33.1 Hz, 2 C), 129.0 (m, 2 CH), 123.4 (q, ^2^*J*_C-F_ = 272.4 Hz, 2 C), 120.3 (dq, ^4^*J*_C-F_ = 7.8, 3.9 Hz, CH), 113.7 (CH_2_), 44.1 (CH_2_), 21.9 (CH_3_). HRMS (ESI +) *m*/*z* calcd for C_12_H_10_F_6_ [M]^+^: 268.0687. Found: 268.0734.

*2-Fluoro-4-(2-methylallyl)-1,1'-biphenyl* (**8**): obtained from [2-fluoro-(1,1′-biphenyl)-4-yl]boronic acid (886 mg) using method b, as a colorless oil (720 mg, 78%), b.p. 170 °C. ^1^H-NMR (CDCl_3_, 200 MHz): δ = 7.59 (d, ^3^*J*_H-H_ = 8.0 Hz, 2H, 2 Ar-H), 7.53–7.32 (m, 4H, 4 Ar-H), 7.05 (t, ^3^*J*_H-H_ = 8.2 Hz, 2H, 2 Ar-H), 4.82 (s, 1H, CH_2_), 4.89 (s, 1H, CH_2_), 3.38 (s, 2H, CH_2_), 1.75 (s, 3H, CH_3_). ^13^C-NMR (CDCl_3_, 75 MHz): δ = 159.7 (d, ^2^*J*_C-F_ = 247.7 Hz, C), 142.4 (C), 141.5 (d, ^4^*J*_C-F_ = 7.6 Hz, C), 135.9 (C), 130.5 (d, ^4^*J*_C-F_ = 4.0 Hz, CH), 129.0 (d, ^5^*J*_C-F_ = 3.0 Hz, 2 CH), 128.4 (2 CH), 127.5 (CH), 126.8 (d, ^3^*J*_C-F_ = 13.5 Hz, C), 124.9 (d, ^5^*J*_C-F_ = 3.2 Hz, CH), 116.4 (d, ^3^*J*_C-F_ = 22.8 Hz, CH), 112.3 (d, ^7^*J*_C-F_ = 32.0 Hz, CH_2_), 44.1 (d, ^5^*J*_C-F_ = 1.4 Hz, CH_2_), 22.1 (CH_3_). HRMS (ESI +) *m*/*z* calcd for C_16_H_15_F [M]^+^: 226.1158. Found: 226.1203.

*1-Methoxy-4-(2-methylallyl)benzene* (**9**) [21]: obtained from (4-methoxyphenyl)boronic acid (775 mg) using method b, as a yellow oil (800 mg, 97%), b.p. 200 °C. ^1^H-NMR (CDCl_3_, 200 MHz): δ = 7.13 (d, ^3^*J*_H-H_ = 8.5 Hz, 2H, 2 Ar-H), 6.86 (d, ^3^*J*_H-H_ = 8.6 Hz, 2H, 2 Ar-H), 4.74 (s, 1H, CH_2_), 4.81 (s, 1H, CH_2_), 3.81 (s, 3H, OCH_3_), 3.28 (s, 2H, CH_2_), 1.69 (s, 3H, CH_3_). ^13^C-NMR (CDCl_3_, 75 MHz): δ = 158.0 (C), 145.6 (C), 131.8 (C), 129.8 (2 CH), 113.7 (2 CH), 111.6 (CH_2_), 55.2 (OCH_3_), 43.8 (CH_2_), 22.0 (CH_3_). HRMS (ESI +) *m*/*z* calcd for C_11_H_14_O [M]^+^: 162.1045. Found: 162.1409.

*1-Methoxy-4-(2-methylprop-1-en-1-yl)benzene* (**9’**) [22]: obtained from (4-methoxyphenyl)boronic acid (775 mg) using method a, as an orange oil (247 mg, 30%), b.p. 225 °C. ^1^H-NMR (CDCl_3_, 200 MHz): δ = 7.10 (d, ^3^*J*_H-H_ = 8.5 Hz, 2H, 2 Ar-H), 6.80 (d, ^3^*J*_H-H_ = 8.6 Hz, 2H, 2 Ar-H), 6.11 (s, 1H, CH), 3.80 (s, 3H, OCH_3_), 1.75 (s, 6H, CH_3_). ^13^C-NMR (CDCl_3_, 75 MHz): δ = 157.7 (C), 137.8 (C), 127.0 (C), 129.8 (2 CH), 113.7 (2 CH), 111.6 (CH), 55.2 (OCH_3_), 22.0 (CH_3_), 19.9 (CH_3_).

*1,2,3-Trimethoxy-5-(2-methylallyl)benzene* (**10**): obtained from (3,4,5-trimethoxyphenyl)boronic acid (869 mg) using method c, as a colorless oil (900 mg, 99%), b.p. 225 °C. ^1^H-NMR (CDCl_3_, 200 MHz): δ = 6.39 (s, 2H, 2 Ar-H), 4.74 (s, 1H, CH_2_), 4.80 (s, 1H, CH_2_), 3.82 (s, 6H, 2 OCH_3_), 3.81 (s, 3H, OCH_3_), 3.24 (s, 2H, CH_2_), 1.67 (s, 3H, CH_3_). ^13^C-NMR (CDCl_3_, 75 MHz): δ = 153.1 (2 C), 144.9 (C), 136.3 (C), 135.4 (C), 112.1 (CH_2_), 105.8 (2 CH), 60.8 (2 OCH_3_), 56.0 (OCH_3_), 45.0 (CH_2_), 22.0 (CH_3_). HRMS (ESI +) *m*/*z* calcd for C_13_H_18_O_3_ [M]^+^: 222.1256. Found: 222.1301. Anal. calcd for C_13_H_18_O_3_ (222.1256): C, 70.24; H, 8.16. Found: C, 69.96; H, 8.01.

*1,2,3-Trimethoxy-5-(2-methylprop-1-en-1-yl)benzene* (**10’**): obtained from (3,4,5-trimethoxyphenyl)boronic acid (869 mg) using method a, as a colorless oil (364 mg, 40%), b.p. 179 °C. ^1^H-NMR (CDCl_3_, 200 MHz): δ = 6.41 (s, 2H, 2 Ar-H), 6.40 (s, 1H, CH), 3.82 (s, 6H, OCH_3_), 3.81 (s, 3H, OCH_3_), 1.77 (s, 6H, CH_3_). ^13^C-NMR (CDCl_3_, 75 MHz): δ = 153.5 (2 C), 144.9 (C), 135.4 (C), 123.7 (C), 115.1 (C), 105.8 (2 CH), 105.2 (CH), 60.8 (2 OCH_3_), 56.0 (OCH_3_), 22.0 (CH_3_), 19.7 (CH_3_).

*(E)-(4-Methylpenta-1,4-dien-1-yl)benzene* (**11**): obtained from (*E*)-styrylboronic acid (607 mg) using method a, as a colorless oil (649 mg, 77%), b.p. 182 °C. ^1^H-NMR (CDCl_3_, 200 MHz): δ = 7.53–7.14 (m, 5H, 5 Ar-H), 6.44 (d, ^3^*J*_H-H_ = 15.8 Hz, 1H, vinylic-H), 6.24 (dt, ^3^*J*_H-H_ = 15.7, 6.8 Hz, 1H, vinylic-H), 4.80 (s, 2H, CH_2_), 2.92 (d, ^3^*J*_H-H_ = 6.7 Hz, 2H, CH_2_), 1.79 (s, 3H, CH_3_). ^13^C-NMR (CDCl_3_, 75 MHz): δ = 144.6 (C), 137.6 (C), 131.4 (CH), 128.5 (2 CH), 128.2 (CH), 127.1 (CH), 126.1 (2 CH), 111.1 (CH_2_), 41.5 (CH_2_), 22.5 (CH_3_). HRMS (ESI +) *m*/*z* calcd for C_12_H_14_ [M]^+^: 158.1096. Found: 158.1459.

*(E)-1-Methyl-4-(4-methylpenta-1,4-dien-1-yl)benzene* (**12**): obtained from (*E*)-(4-methylstyryl)boronic acid (664 mg) using method b, as a colorless oil (650 mg, 92%), b.p. 152 °C. ^1^H-NMR (CDCl_3_, 200 MHz): δ = 7.28 (d, ^3^*J*_H-H_ = 8.2 Hz, 2H, 2 Ar-H), 7.12 (d, ^3^*J*_H-H_ = 8.0 Hz, 2H, 2 Ar-H), 6.41 (d, ^3^*J*_H-H_ = 15.8 Hz, 1H, vinylic-H), 6.19 (dt, ^3^*J*_H-H_ = 15.8, 6.9 Hz, 1H, vinylic-H), 4.81 (s, 1H, CH_2_), 4.91 (s, 1H, CH_2_), 2.91 (d, ^3^*J*_H-H_ = 6.8 Hz, 2H, CH_2_), 2.35 (s, 3H, CH_3_), 1.78 (s, 3H, CH_3_). ^13^C-NMR (CDCl_3_, 75 MHz): δ = 144.7 (C), 136.8 (C), 134.9 (C), 131.3 (CH), 129.2 (2 CH), 127.2 (CH), 126.0 (2 CH), 112.0 (CH_2_), 41.5 (CH_2_), 22.5 (CH_3_), 19.5 (CH_3_). HRMS (ESI +) *m*/*z* calcd for C_13_H_16_ [M]^+^: 172.1252. Found: 172.1295.

*(E)-1-(4-Methylpenta-1,4-dien-1-yl)-4-(trifluoromethyl)benzene* (**13**): obtained from (*E*)-[4-(trifluoromethyl)styryl]boronic acid (886 mg) using method b, as a colorless oil (820 mg, 88%), b.p. 179 °C. ^1^H-NMR (CDCl_3_, 200 MHz): δ = 7.55 (d, ^3^*J*_H-H_ = 8.4 Hz, 2H, 2 Ar-H), 7.45 (d, ^3^*J*_H-H_ = 8.3 Hz, 2H, 2 Ar-H), 6.47 (d, ^3^*J*_H-H_ = 16.0 Hz, 1H, vinylic-H), 6.33 (dt, ^3^*J*_H-H_ = 15.8, 6.2 Hz, 1H, vinylic-H), 4.79 (s, 1 H, CH_2_), 4.83 (s, 1H, CH_2_), 2.93 (d, ^3^*J*_H-H_ = 6.2 Hz, 2H, CH_2_), 1.79 (s, 3H, CH_3_). ^13^C NMR (CDCl_3_, 75 MHz): δ = 143.9 (C), 142.3 (C), 131.1 (CH), 130.2 (CH), 128.9 (q, ^3^*J*_C-F_ = 32.4 Hz, C), 126.2 (2 CH), 125.5 (q, ^4^*J*_C-F_ = 3.8 Hz, 2 CH), 124.3 (q, ^2^*J*_C-F_ = 271.7 Hz, C), 112.0 (CH_2_), 41.4 (CH_2_), 22.5 (CH_3_). HRMS (ESI +) *m*/*z* calcd for C_13_H_13_F_3_ [M]^+^: 226.0969. Found: 226.1015.

### 3.3. Synthesis of 2,4,6-tri-p-Tolyl-1,3,5,2,4,6-trioxatriborinane

*p*-Tolylboronic acid (1 g) in methallyl alcohol was heated for 1 h at 120 °C, under microwave irradiation. After cooling the reaction mixture, water was added and a white precipitate appeared: it was filtered off and recrystallized in cold water. The solid was then dried in an oven for 24 h. Product was obtained as a white solid (boronic acid conversion = 43%).

*2,4,6-tri-p-Tolyl-1,3,5,2,4,6-trioxatriborinane* (**14**) [5] m.p. 263 °C (259–261 °C)^5a^. ^1^H-NMR (CDCl_3_, 200 MHz), δ = 8.13 (d, ^3^*J*_H-H_ = 7.9 Hz, 6 Ar-H), 7.31 (d, ^3^*J*_H-H_ = 7.9 Hz, 6 Ar-H), 2.44 (s, 3CH_3_). ^13^C-NMR (CDCl_3_, 75 MHz), δ = 142.96 (3 C), 135.73 (6 CH), 133.56 (3 C), 128.80 (6 CH), 21.94 (3 CH_3_).

*1-Methyl-4-(2-methylallyl)benzene* (**15**): obtained from 2,4,6-tri-p-tolyl-1,3,5,2,4,6-trioxatriborinane (1450 mg) using method b (18 mg, 3%), as a colorless oil, b.p. 177 °C. ^1^H-NMR (CDCl_3_, 200 MHz), δ = 7.10 (s, 4 Ar-H), 4.74 (s, 1H, CH_2_), 4.80 (s, 1H, CH_2_), 3.29 (s, CH_2_), 2.34 (s, CH_3_), 1.69 (s, CH_3_). ^13^C-NMR (CDCl_3_, 75 MHz), δ = 145.4 (C), 136.7 (C), 135.5 (C), 129.0 (2 CH), 128.8 (2 CH), 111.7 (CH_2_), 44.3 (CH_2_), 22.1 (CH_3_), 21.1 (CH_3_). HRMS (ESI +) *m*/*z* calcd for C_11_H_14_ [M]^+^: 146.1096. Found: 146.1141.

## 4. Conclusions

The route described herein for the synthesis of allyl alkenes remains very efficient. Not only does it open the way to a wide range of boronic acids, but, also, as these investigations on new reaction conditions illustrate, it can be carried out with unstable boronic acids subject to deboronation or isomerization. Moreover, this kind of cross-coupling reaction allows stereoselectivity to be maintained. Our mechanistic study highlights the importance of the Lewis acidity of the boronic acid used. Moreover, when protic solvents and unreactive methallyl alcohols are used in such cross-coupling reactions, adding a base could increase reactivity.
